# Medical students’ perceptions of stress due to academic studies and its interrelationships with other domains of life: a qualitative study

**DOI:** 10.1080/10872981.2019.1603526

**Published:** 2019-04-22

**Authors:** Christin Bergmann, Thomas Muth, Adrian Loerbroks

**Affiliations:** a Institute of Occupational, Social and Environmental Medicine, Center for Health and Society, Faculty of Medicine, University of Düsseldorf, Düsseldorf, Germany; b Mannheim Institute of Public Health, Social and Preventive Medicine, Mannheim Medical Faculty, Heidelberg University, Heidelberg, Germany

**Keywords:** Germany, medical students, psychological stress, well-being, work-family conflict

## Abstract

**Background**: Medical students have been found to experience considerable stress due to their academic studies. The high demands associated with academic studies may interfere with demands in other domains of life. Conversely, demands in those other domains of life may conflict with academic studies.

**Objective**: We aimed to better understand the potential inter-relationships between the demands related to academic studies and in other domains of life.

**Design**: A total of 68 medical students from a medical school in Germany participate in eight focus groups. Sessions were structured by a topic guide and were recorded, transcribed and content-analyzed.

**Results**: Embarking on one’s medical studies was perceived to be associated with important personal challenges, such as living alone for the first time and finding a new social role in one’s peer group. Permanent stress was perceived to result in emotional exhaustion, which spilled over in other domains of life. Students reported to feel guilty if they did not spend their limited spare time on learning. Consequently, they felt to have little time for leisure time activities and a healthy lifestyle. Feelings of social isolation, especially during exam phases, were reported. Leisure activities were perceived to facilitate recovery from academic stress. Social ties were subjectively able to reduce stress, but also to increase stress due to demands. Side jobs were perceived to increase stress and to be time-consuming and were thus perceived to result in poorer academic performance. Certain personality characteristics seemed to amplify those perceptions. For instance, high levels of conscientiousness were felt to relate to higher expectations regarding one’s academic performance and thus increased stress.

**Conclusion**: The demands associated with medical studies, the demands in private life, lacking resources for recovery and certain personal traits as well as interactions between those domains can contribute to stress among medical students and reduce well-being.

## Introduction

The health and well-being of medical students is an issue which has attracted increasing research interest. The available evidence suggests that medical students’ health status is poorer than that of the general population [1–4]. This holds in particular true for medical students’ mental health [5–9]. For instance, the prevalence of depression in this population has been estimated at about 40% [6,9]. Academic stress has been identified as an important predictor of poor mental health [10]. Also, stress is an important cause of declining empathy among medical students [11,12]. This is further aggravated by the observation that every fifth medical student at the beginning of his/her study shows excessive commitment and propensity to exhaustion [13]. Medical studies are perceived to be characterized by competition, lacking time for leisure activities or social contacts and schedules that demand exclusive dedication, all of which can lead to reduced life satisfaction [14–16]. To help medical students cope with stress and to reduce the likelihood of unfavorable health-related outcomes, interventions have been devised covering, for instance, relaxation techniques and stress management techniques [17]. Overall, stress levels and well-being among medical students and approaches to facilitate coping with stress have been repeatedly researched [1,18–20].

While their academic studies likely represent a very important domain of medical students’ life, there are additional domains of life (e.g., family, friends, leisure-time activities). It seems plausible that these non-academic domains of life interact with academic stress. For instance, social support from family members may help to reduce stress or may buffer against the sequels of stress [21]. This study sought to expand the current research on stress among medical students by exploring the interrelatedness of the demands due to medical studies with other domains of life, which may be associated with competing demands or that may offer coping resources. Doing so, the present study builds on models of work-family-conflict, which have been established in research among adult working populations. A work-family conflict may be experienced, for instance, when time devoted to the requirements of one role makes it difficult to fulfill the requirements of another role (time-based conflict) [22]. In terms of medical studies, a time-based conflict may imply that students feel to lack time for leisure time activities due to spending most of their time on medical studies, which implies potentially reduced opportunities for recovery. Previous studies have found for instance that socializing with peers decreases during medical school [23]. Consequently, social support resources are reduced and loneliness is another stressor for medical students [24]. Another type of conflict is a strain-based conflict which exists when strain caused by one role affects another role [22], for instance, if medical students are exhausted from studying and feel emotionally too drained to participate in family activities.

We set out to explore the possible interrelatedness of medical students’ demands in academic life and in their non-academic life. While there are studies that have addressed this issue broadly (e.g., a study showing that pursuit of a medical career is associated with less life satisfaction [25]), research which comprehensively explores the specifc interrelatedness of stressors pertaining to medical students’ academic life and their non-academic life is lacking to the best of our knowledge. We aimed to address this knowledge gap to inform the development of potential interventions that may assist students in reducing conflicts between those major domains of their life and to thereby potentially improve the mental health of this population [26].

## Materials and methods

### Research team

The research team consisted of two medical students (CB and StS, see Acknowledgments), and two researchers with frequent teaching contact to medical students, with experience related to the facilitation of focus groups, and expertise in qualitative research and stress research (TM and AL).

### Participant recruitment

A convenience sample of students from the medical school at the University of Düsseldorf, Germany, was recruited for focus groups through social media or was personally approached by the two research team members who were also medical students. We sought to recruit entire groups of students who had been taking the same course. This was done, because we expected that there was likely established trust within those groups, which may promote open discussions. Participation was voluntary and was rewarded with cinema tickets or vouchers for a bookshop. The only selection criterion was current enrollment as a medical student. We specifically recruited students from both earlier stages of the medical education (i.e., term 2–4) and more advanced stages (i.e., term 9–10) to increase a likelihood that the full range of potential views and experiences is shared in the focus groups.

### Data collection

The research team created a topic guide for the focus groups. The topic guide consisted of four overarching questions with potentially further sub-questions. First, a broad introductory question was supposed to initiate an open discussion and to encourage every participant to share his/her thoughts early on. The subsequent block of questions addressed perceived stress and psychological resources. Issues relating specifically to stressors and coping are not at the core focus of the current manuscript and will be reported elsewhere. The next block of questions touched on possible interrelationships of academic studies and potentially competing demands in other domains of life (If so, which interrelationships exist and what are reasons for it? How do you handle them?). In this block of questions, bi-directionality was explored (i.e., effects of academic studies on other domains of life and effects of other domains of life on academic studies). The information collected by those questions constitutes the main data for the present report. The final question related to the discussion of interventions, which may help to reduce academic studies. Overall, eight focus groups were held between November 2013 and July 2015  and were all facilitated by the same senior researcher (TM), who has profound expertise related to the facilitation of focus groups. Discussions were digitally recorded and transcribed.

### Data analysis

Content analysis *was carried out according to an approach suggested by Mayring* [27] using the MaxQDA 12 software package. Specifically, initial overarching codes (i.e., deductive codes) were used to capture the main issues that were raised based on the topic guide. These codes (i.e., effects of academic studies on private life; effects of private life on academic studies) were then further specified, that is, additional codes and subcategories were created based on the data (i.e., inductive coding). The assembled statements within and across categories were repeatedly reviewed to examine to what extent categories appeared to be internally consistent, but also conceptually different from other categories.

We considered each participant statement equally important irrespectively of the statement's frequency. We aimed to explore the full scope of potential views and to find new insights to emerge (i.e., conventional content analysis) rather than to quantify the frequency of statements (e.g., like in summative content analysis) [28].

The material was initially coded by both CB and AL and the resulting codes were discussed to establish an initial coding framework, which was to be used for subsequent rounds of content analysis. Categories were discussed, for instance, in terms of their potential redundancy (selectivity of categories), comprehensibility (logical allocation of main categories and subcategories) and replicability (e.g., the extent to which AL would create a similar code structure) until an agreement was reached. CB coded all transcripts again, and CB and AL discussed the resulting codes and code structure. Afterwards, a third round of coding was performed by CB, which was considered final.

For the purpose of presenting our observations, the data will be organized by three sections. For each section, quotes that illustrate our observations will be presented in tables in the results section (see Tables 1-3). The first section addresses the impact of academic studies on private life. The second covers the impact of private life on academic studies. A final section details the role of personality, which initially emerged inductively from our data. In subsequent rounds of coding, we then applied deductively the Big Five Model [29] as a coding framework. The Big Five Model is a taxonomy of five personality traits; openness to experience, conscientiousness, extraversion, agreeableness, and neuroticism [30]. Individuals can be classified in terms of the extent to which each trait is accentuated. Different levels of those traits may predict, for instance, how one responds to and copes with stress [31]. Certain personality traits may further be particularly relevant for medical students due to how those students are selected: there are multiple routes to one’s admission for medical schools in Germany: i) selection based on the best Abitur^1^ grades in state ranking, ii) waiting time (i.e., students who waited since their Abitur; at current the required waiting time equals 7.5 years) and iii) internal selection processes of medical schools which may rely on Abitur grades and/or one’s passing of an exam or personal interviews [32]. However, the majority of medical students is chosen by Abitur grades [33] and thus academic performance. Thus, individuals with personality traits that are positively associated with academic performance may be more likely to be admitted (see Discussion section).

**Table 1.** Quotes illustrating the perceived effects of academic studies on one’s private life and associated personal challenges.

A1	During the first semester, just mentally (,) I think we had to take eleven exams (.) and everything else and, er, I was completely exhausted. I couldn’t eat, couldn’t sleep, I was, well, I really was a nervous wreck.
A2	But somehow my whole life is centred around studying. In my spare time, I hardly do anything but study. (.) And things like exercising once a week, I don’t even begin to do this because I think then I will miss out on studying for yet another day. That’s the only thought one has.
A3	And each time I do something else than studying I have a bad conscience because I ought to study. And each time I study I feel terribly stressed because I think I need a break. A kind of balance develops because you feel bad when you study and you feel bad when you don’t. It’s like that all the time.
A4	We even studied during the Christmas break. With a heavy heart, but still. You visit some relatives and take along your flashcards. (.) You sit on the sofa with earplugs in your ears, but at least you stayed with your relatives.
A5	This is simply the pressure one feels, that you know that if you fail three times, and that happens really, really quickly, then you will never be able to do it again. (.) I don’t know what else I would do with my life and if I failed three times and it was over then (.) my life would be in shambles.
A6	Well I think there are a lot of people who, er, also a lot of drop-outs who are deeply disappointed and exhausted, particularly in the pre-clinical phase, because there is so much stress and pressure that they can’t cope with. (.) I believe there are some people who don’t break down but who feel it impacted their personality.
A7	It happens every semester (.) I went through some phases, I had never experienced this before, I was just lying there and crying and I thought I really can’t, I can’t go on anymore. That was really, really, really exhausting. (.) I don’t know if I will succeed.
A8	What is quite severe for me, well I think that a student of medicine who lives alone, that’s at least my experience, will become quite lonely when preparing for exams. (.) I lived alone during the first two semesters and when we had exams I went shopping at 11:30 at night, because the one particular grocery store was open until midnight, the shop I frequented was a bit further away, but there was always a student working as a cashier, (.) I could chat with her. I always found that a bit sad because if I have time now (,) and then I went shopping last night again just to meet another person. That really wasn’t, er, quite nice.
A9	I’m almost finished and I derive a certain kind of self-confidence from this. If my studies didn’t break me then it doesn’t matter what else is going to happen, more or less. I will be able to cope with other things as well, I will be able to handle other things successfully.

**Table 2.** Quotes illustrating the perceived effects of one’s private life on academic studies and associated personal challenges.

B1	When worse comes to worse, sure, everyone feels low sometimes, then I always call my parents (.), because it’s such a great support.
B2	It also happens that my mother comes to my room when I want to study, and then she wants to chat with me for half an hour, you can’t just ask her to leave. Therefore, one gets easily distracted by so many different things.
B3	I am not as close to my old friends as I used to be, they all study (.), but they went on holiday last week and I couldn’t join. (.) But on the other hand, I found a lot of new friends here and therefore I don’t perceive this as very troubling.
B4	On the other hand, I’m glad that I have some very good friends here who also study medicine, whom I can tell, 'well, I couldn’t cope ‘well with the autopsy, how about you?' (.) In a way this is important, because my boyfriend or other people don’t understand it. I tell them a person had this and that and it takes a long time to explain what that is and so you have, people with a medical background, you can tell them that you felt distressed by something and perhaps they feel the same.
B5	I think that because studying takes so much time and one spends a lot of time at university and is only around people from university. It’s the only subject to talk about. No matter where you go. There are always some students of medicine. (.) Then you talk about university. Then perhaps you feel stressed out by someone else because he’s better at something than you are. That there’s nothing but medicine in my life. That’s terrible. Somehow you don’t know how to change it, because you always have to study. And never have time to meet with friends.
B6	What really makes me terribly sad is that when I call my friends (.) and then they tell you, I’m going out tonight with this friend and yesterday I met for coffee with another friend and now we plan on going shopping tomorrow. (.) And you sit there and think, great, I can’t do any of these things. That makes me terribly sad.
B7	A couple of weeks ago I had to work all night for four days and it was always the same thing, get up in the morning and go to university, then skip studying because one’s too tired, and then another course. Half asleep. Then go to work in the evening.
B8	Work can really be a problem. For example, this month I can’t work half of the month because of exams, because sometimes I need time for studying, and then I don’t have enough money.
B9	I used to have about 10 different hobbies. And every day there was another activity I participated in. (.) And here you basically have nothing besides university.

**Table 3.** Quotes illustrating the role of personality in the interrelatedness of one’s academic studies and private life.

C1	Well, when you think 'OK, I will pass the exam regardless of the result', then you feel less stressed as when you aim at achieving a good result. (.) I think much depends on what you expect from studying and on how much one expects from oneself, and that’s perhaps what adds to or distracts from the stress.
C2	That’s like another type of school with tests, but for people (.) who tend to procrastinate. And the number of things left undone keeps growing and growing and then one day it’s shit, I still have to do all of these things.
C3	Especially during the preclinical phase, I can make a decision, either I want some kind of private life or I will excel at university, one of these two.
C4	But I really feel so bad that I don’t sleep well quite often. That in the last one or two weeks I have recurring dreams of standing in the autopsy room and have exams. It sounds ridiculous but it’s a real burden for me.

## Results

The eight focus groups – each lasting for roughly 90 min – were held with 68 medical students. The size of the focus group ranged between six and eleven medical students. There was one exception, that is, a very small group comprising only two participants. Data on age and sex were collected among participants in the last six focus groups. The mean age was 24 years (ranging from 18 to 34 years) and 77% of the participants were female. In total 42% of participants were in early stages of their medical study (i.e., term 2–4) and the remainder in advanced stages (i.e., term 9–10 semester).

### Perceived effects of academic studies on private life and personal challenges

#### New living arrangement

Many participants expressed that their transition from school to university was associated with major personal challenges. Those were in particular the organizational challenges related to living alone for the first time (e.g., what to do when the internet connection at home breaks down).

#### Losing the position at the top of one’s class

Most of the students had previously belonged to the top performing students at their respective schools in terms of their grades. As the demands in medical school are higher and as peer students are just as performance-oriented and motivated, this top position may be lost. Such loss was associated with self-doubt regarding one’s intellectual abilities, especially when students felt that they do their best. Some participants experienced resentment among fellow students due to pronounced competition.

#### Medicine becoming the main/only domain of life

Students perceived that especially the first period of their study is characterized by constant stress (see quotation A1 in Table 1). As a consequence, students reported to gradually neglect almost any other domain of life and therefore medical studies were felt to become the only domain in their life (A2), which was found to be saddening.

#### Guilty conscience and fear of failure

Many participants mentioned a time-based conflict. Students reported to have a guilty conscience whenever they did not spend their limited spare time on studying (A3). This bad conscience spilled over into formal holidays (A4). This was subjectively fostered by the feeling to be never finished with studying. At medical schools in Germany, repeated failure at exams (i.e., three consecutive failures on the same exam) results in students being expelled from medical studies at any medical school in Germany. This feature is associated with major fear among the majority of students (A5).

#### Structural aspects

Students’ opportunities for recovery during formal semester breaks were perceived to be very constrained. Moreover, the structure of the medical curriculum does not allow for any extended absence (e.g., due to sickness), if one seeks to complete one’s studies in the regular time period. This was felt to be very distressing. An expanded period of studies can lead to higher financial strain.

#### Feelings of permanent stress

Students reported to feel permanent stress, which was perceived to result in emotional exhaustion or thoughts about quitting medical studies (A6 and A7). Furthermore, participants felt that their stress may contribute to unhealthy lifestyles, such as skipping meals, lack of exercise or a lack of sleep. Some participants reported social isolation during exam phases (A8). Many participants alluded to a seeming paradox, that is, that medical studies intend to teach students how to cure people, but this is taught in a way, which jeopardizes the students’ own health.

#### Positive effects

Many students seemed to be grateful for their own fairly good health due to their daily encounters with patients’ suffering and death. For some students, this experience puts their own day-to-day problems into perspective. Participants mentioned that if one successfully masters their medical studies, this experience could contribute to a sense of self-esteem, self-efficacy, and resilience (A9).

### Effects of private life on academic studies and personal challenges

#### Family

Family ties were perceived by many students as important support resources that buffer against permanent stress (B1, see Table 2). Several participants viewed their family, as a corrective whenever they lost confidence into their academic ability, e.g., due to failure to be at the top of one’s class despite best efforts. Living with one’s family was felt to be protective against social isolation, but at the same time family members were perceived to contribute to stress in case they make requests for time (B2).

#### Friendship

Contact with friends who are not involved in medical studies may be reduced while ties are established with fellow medical students (B3). According to some participants ‘non-medical’ friends (e.g., from schooldays) cannot fully relate to their current situation and may show limited understanding (e.g., if appointments are frequently canceled), which leads to a reduction or termination of those ties or to conflicts. By contrast, friends who are fellow medical students seem to better understand students’ stress and show empathy in challenging academic situation (B4). The perceived downside to close ‘medical friendships’ was the risk that all conversations are usually narrowed down to medical studies (B5). Therefore, many students also appreciated ‘non-medical friends’ because spending time with those friends is usually unconnected with medicine. Several participants expressed to have far less time for leisure activities as compared to ”non-medical” friends, which can be saddening (B6).

#### Side job

Some students need to take a side job to be able to cover their living expenses. Such a side job in addition to academic studies may contribute to further stress and was felt to result in poorer academic performance as there is less time for studying (B7). The demands of one’s medical studies and one’s side job were perceived to induce time-based conflicts. Importantly, if medical studies were prioritized the limited time for the side job (and thus lower income) induced financial strain (B8). A side job carried out in a medical field appeared to have the potential to spur one’s motivation for medical studies and to provide reinforcement of one’s career choice. It appeared to be challenging for many students though to find a needed side job because of inflexible short-term scheduling in the medical curriculum.

#### Leisure activities

Some participants succeeded in deliberately taking time for leisure activities, especially for those unassociated with medicine, such as sports. Making this time available was however felt to come at the cost of jeopardizing one’s academic performance because of less time for learning. For this reason, some students drop their leisure time activities, which they used to enjoy (B9).

### Personality

The relevance of personality traits to the interrelatedness of medical studies and other domains of life emerged as an additional key theme from our focus groups. Personality may be conceptualized in terms of the Big Five Model [29] (i.e., conscientiousness, agreeableness, extraversion, neuroticism, and openness to experience). We used this model to structure our analyses and will use it to report our observations. Our findings can be related to four of the five personality traits (the exception being openness to experience):

#### Conscientiousness (i.e., the tendency to be dependable and self-disciplined)

Study participants felt that high levels of conscientiousness were associated with higher stress levels during medical studies due to high expectations regarding one’s academic performance (C1, see Table 3). As a consequence, several participants expressed to spend their daytime almost exclusively on studying and prioritized academic demands above social relationships or any leisure activity. By contrast, though a very low level of conscientiousness was perceived to promote stress in terms of procrastination, i.e., the postponement of exams or exam preparations with the consequence of ultimately needing to attend additional exams during a given period of time which implies that there is overall less preparation time for each exam (C2).

#### Agreeableness (i.e., the tendency to be cooperative and not competitive)

Participants who made statements suggestive of low agreeableness and thus pronounced competitiveness appeared to struggle harder with the loss of their top performance rank.

#### Extraversion (i.e., tendency to be sociable, outgoing and energetic)

Some students reported to feel that they have to choose at the beginning of academic studies between either a private life in addition to medical studies or success in medical studies (C3). The choice to prioritize studies may be especially difficult for students with a high level of extraversion because they tend to suffer more from social isolation (B6).

#### Neuroticism (i.e., tendency to be sensitive or prone to psychological stress)

Presumably high levels of neuroticism (in terms of anxiety and self-doubt) may contribute to the perception of stress and difficulties in coping with stress (C4). Furthermore, several participants reported to feel unable to enjoy leisure time, because of the above-mentioned feelings of guilt. This may be the result of a combination of high levels of both neuroticism and conscientiousness.

## Discussion

We found that the demands or stress associated with academic studies exert unfavorable effects on private life and wellbeing (i.e., reduced social ties, leisure activities and losing the position at the top in terms of grade ranking). At the same time though mastering medical studies can enhance self-esteem, self-efficacy, and resilience. However, non-academic life can also be stressful (i.e., unmet social expectations or financial worries). Specific aspects of one’s non-academic life may provide valuable resources to buffer against stress experienced in medical studies (e.g., social networks). Some personality characteristics – which we conceptualized in terms of the Big Five model – may affect the perception of stress due to academic life and due to non-academic life and of both domains' interrelatedness.

### Findings in light of the literature

#### Effects of academic studies on private life and personal challenges

In line with earlier research, our study suggests that the beginning of academic studies may be a particularly stressful new stage of life (i.e., social and organizational challenges) for some participants [2,34]. In terms of personal challenges, the failure to achieve previous school performance levels was reported as distressing by our study participants. Among some students, the loss of this important self-defining feature seemed to induce self-doubt and anxiety, which is in agreement with findings from earlier research [2,35].

Academic studies may be associated with chronic and high stress exposure, and prior evidence has shown that such stress is linked to worse performance, poorer satisfaction, intentions to quit, and elevated depression, anxiety, higher risk of suicidal ideation or physical problems [25,34,36–39]. As a consequence of chronic exposure, several students suffer from poor health and fatigue [34,37], which is in line with our observation. In our study, some participants framed this experience as a paradox, that is, the fact that they study a medical subject with the aim to cure people, but that this study jeopardizes their own health, which is supported by further observations [40].

Research suggests that the demand for increased time for private life gains relevance among medical students [41,42]. In terms of work-family conflict models, several participants from our study need to deal with the work-family-conflict type “overload“, which reflects that the total demand on time is too great to meet the demands of different roles comfortably [43]. Just like in our study, several participants feel that they need to choose between private life or studies and not every medical student strives at all cost for her/his career [25,41,42,44]. A cross-sectional study from Germany suggested that more than one-third of medical students report not to have time to pursue individual interests [3]. Our study confirms this overall perception and further adds that many participants may in fact neglect almost all activities except academic studies. Consequently, medical studies subjectively become the only domain of life. Supporting this notion, a US study found that socializing decreases in medical school [23]. It has also previously been observed that insufficient time for social demands can enhance perceived stress and may lead to a sense of guilt [35], which is in line with findings from our study.

#### Effects of private life on studies

Our study suggests that private life (i.e., social ties and leisure activities) could be an important resource for handling study demands. These findings are in line with the results of earlier studies showing that social support and regular exercise are associated with lower psychological distress or rather better quality of life among medical students [35,38,45]. A New Zealand study showed that ‘medical friendships’ are helpful in adjusting to medical school due to emotional support and/or a shared understanding of academic structures [46]. Our study confirms this and adds that medical students may also wish for and benefit from spending time with ‘non-medical friends’, as medical studies are not a prominent topic in those friendships. This feature may succeed in temporarily distracting one’s focus from academic studies and may thus facilitate recovery.

A cross-sectional study from Germany suggested that the experience of time pressure is more associated with medical studies rather than with one’s side job [47]. By contrast, participants from our study expressed that side jobs could be perceived as additional strain as they are time-consuming. Also, whenever side jobs cannot be taken to the required extent to cover expenses, financial worries may emerge. An Australian study confirmed that there is a positive correlation between financial worries and perceived stress [38].

#### Personality

We found that specific personality traits may affect the perception of academic stress among medical students, such as a high degree of conscientiousness. Studies from Germany have found that many medical students exhibit personality traits (i.e., poor emotional stability or a high sense of dominance), which are associated with health problems [13,48]. This is in line with our observation that a high degree of conscientiousness may contribute to higher stress levels. Positive aspects of a high degree of conscientiousness were also shown by earlier research, which reported that high conscientiousness correlates with better performance [49,50]. As described in the materials and methods section the majority of medical students is chosen by Abitur grades and thus by their academic performance. High levels of conscientiousness are related to better academic performance [51]. Consequently, individuals with high conscientiousness may be more likely to be admitted. However, high conscientiousness also predicts stress [52], which may contribute to poorer health [53,54]. Therefore, the current approach in Germany to the selection of students for (i.e., building heavily on grades) favors the selection of students with strong academic abilities who may at the same time be vulnerable to stress and subsequently poor health. A potential selection of medical students towards high levels conscientiousness may also translate into a different personality trait later in the medical career: conscientiousness is related to an exaggerated sense of responsibility, which in turn is one aspect of compulsiveness in the personality of physicians [55]. Thus, students’ conscientiousness may partly explain subsequent compulsiveness once they are trained physicians.

### Methodological considerations

Some potential methodological limitations deserve mentioning. First, it cannot be ruled out that our approach to participant recruitment is susceptible to selection bias. For instance, very dissatisfied (or very satisfied) students may have been more likely to participate and this could have limited the scope of opinions that were shared. Further, 77% of the participants were female. If there were gender differences related to our research question, views of male students could be somewhat under-represented in our study. It deserves mentioning though that the over-representation of female students in our study partly matches the actual gender distribution among medical students at the University of Düsseldorf (i.e., the proportion of female students raged from 62% to 64% between 2011 and 2019). Second, the first author (CB) herself was a medical student at the time of data collection and data analysis. Although the subjective interpretation of data is inherent to qualitative research, some of the first author’s prior experiences as a medical student may have overly guided her interpretation of the material. This issue was addressed by the fact that transcripts and data analyses were reviewed by and discussed with AL, who is a public health researcher. Further, the focus groups were not facilitated by CB, but by TM (a psychologist) to reduce potential bias during data collection. Third, group discussions were held until thematic saturation of topics was achieved. While focus groups are particularly useful to explore issues in depth, anonymity is not given – especially if group members know each other – and therefore sensitive topics were possibly not raised (e.g., alcohol consumption) or not shared in full depth (e.g., severely impaired mental health). Finally, we recruited two subpopulations of medical students who were either in earlier stages of their medical study (i.e., term 2–4) or in advanced stages (i.e., term 9–10). The aim of our study was *not* to examine whether the interrelatedness of academic stressors and stressors in other domains of life differed by the year of medical studies. One may speculate though that the reporting or experience of the issues covered by our study is affected by the academic year. For instance, the reporting of students in earlier stages may be affected not only by their current stressors but also by anticipated stressors. By contrast, students in advanced stages are apparently selected towards their ability to have successfully coped with previous stressors. This sense of mastery may have affected their reporting (i.e., toning down the experience of current and/or past stressors).

### Implications

This study suggests that studying at medical schools can exert negative effects on one’s private life in terms of lacking time for recovery (i.e., in terms of leisure activities or social contact) and poor mental health. Individual-level preventive action may build on compulsory stress management training, which needs to be a part of the curriculum and thus cannot be skipped due to a sense of time pressure. The literature suggests effective approaches to reduce perceived stress, such as stress management training involving mindfulness-based stress reduction (13, 20, 37), autogeneous training [56] or biofeedback [57]. Some participants express to have a guilty conscience because of the feeling that they do not study hard enough. This could be addressed by providing an inventory detailing academic expectations, for instance, with regard to the topics that should be covered by students (e.g., on a monthly basis). Also, unrealistic expectations related to one’s performance in a highly committed and skillful peer group should be reflected early on. Curricula can induce a sense of high stress when full attendance is required implying that absence is not possible and/or cannot be compensated. Introduction of new learning technologies including e-learning [58] and virtual patient simulation [59] could be opportunities to create a curriculum which caters for the needs of individual students [60]. Furthermore, some participants reported to be distressed by their failure to achieve top performance levels in terms of grades. This may result in excessive work commitment. One approach to reduce this stressor would be to avoid grading whenever possible and to only inform students about whether they have passed or not.

As mentioned above, we suspect that the selection of students for medical education in Germany favors students with personality traits that are associated with better academic performance, but also with impaired health [13]. This issue seems to be addressed to some extent as the selection procedures currently need to be revised in Germany to ensure that less emphasis is placed on grades at school [61].

### Conclusions

We found that academic studies may lead to various personal challenges (e.g., living far away from home) and affects private life in multiple ways. Negative effects may be less time for social contacts and leisure activities. Private life can help students to cope with academic stress. Social contact could lead to an additional burden if social requirements cannot be fulfilled by students. Personality traits may modify the perception of and responses to stress.

## Data Availability

Data can be obtained from the corresponding author for research purposes upon reasonable request.

## Note

1. Abitur = the general qualification of university entrance, which is similar to the American Grade Point Average.
